# Silver Inhibits *Lemna minor* Growth at High Initial Frond Densities

**DOI:** 10.3390/plants12051104

**Published:** 2023-03-01

**Authors:** Indigo T. Tran, Jordan A. Heiman, Victoria R. Lydy, La Toya Kissoon

**Affiliations:** 1 Department of Biology, Missouri State University, Springfield, MO 65897, USA; 2 Department of Biology, The University of Mississippi, University, MS 38677, USA; 3 Department of Biological Sciences, Arkansas State University, Jonesboro, AR 72401, USA

**Keywords:** *Lemna minor*, frond density, silver nanoparticles, silver nitrate

## Abstract

Silver nanoparticles (AgNPs) are the most popular engineered nanomaterials in consumer products due to their antimicrobial properties. They enter aquatic ecosystems via insufficient purified wastewaters from manufacturers or consumers. AgNPs inhibit growth of aquatic plants, including duckweeds. Growth media nutrient concentration and initial duckweed frond density can affect growth. However, it is not well understood how frond density affects nanoparticle toxicity. We investigated the toxicity of 500 µg/L AgNPs and AgNO_3_ on *Lemna minor* at different initial frond densities (20, 40, and 80 fronds per 28.5 cm^2^) over 14 days. Plants were more sensitive to silver at high initial frond densities. Growth rates based on frond number and area were lower for plants at 40 and 80 initial frond density in both silver treatments. AgNPs had no effect on frond number, biomass, and frond area at 20 initial frond density. However, AgNO_3_ plants had lower biomass than control and AgNP plants at 20 initial frond density. Competition and crowding at high frond densities resulted in reduced growth when silver was present, therefore plant density and crowding effects should be considered in toxicity studies.

## 1. Introduction

Silver nanoparticles (AgNPs) are popular nanomaterials in consumer products due to their antibacterial properties and multifunctional applications [1,2]. AgNPs can disrupt wastewater treatment processes, such as microbial growth and nitrification [3,4], when released in wastewaters from manufacturers and consumers [5,6,7] and discharged into aquatic ecosystems [8,9,10]. Once in aquatic ecosystems, AgNPs pose threats to ecosystem processes and aquatic organisms. In plants, exposure to AgNPs inhibited growth and decreased photosynthetic rates, chlorophyll production, and germination rates [11,12,13,14].

Duckweeds are ideal for studies on nanoparticle toxicity because of their small size, fast growth rates, ease of harvest, and ability to reproduce vegetatively [15]. These characteristics make duckweeds ideal candidates for phytoremediation of wastewaters [16,17,18,19]. Table 1 summarizes the toxic effects of AgNPs on duckweeds, which depended on plant species, nanoparticle size, concentrations, and exposure times. These toxic effects included chlorosis, oxidative stress, and decreased frond number, biomass, and growth rates.

Inhibition test guidelines recommended frond number as the primary measurement variable for assessing toxicity of substances to *Lemna* sp., with at least one other variable such as frond area, dry weight, or fresh weight [30]. Table 2 lists initial frond numbers reported in studies assessing the effects of AgNPs on duckweeds. Toxicity effects were assessed on frond number, dry weight, and frond area. Some studies assessed one or two growth measurements and none assessed all three. Frond number and dry weight were popular measurements, with few studies reporting effects on frond area (Table 2). Some studies in Table 2 did not provide their initial frond numbers or used low initial frond numbers (<21). Studies that did not clearly state initial frond numbers reported effects on frond numbers, but this is difficult to interpret and compare with our findings.

While several studies relied on frond number as a primary growth measurement, none addressed the effects of nanoparticle toxicity on initial frond density. Differences in toxicity effects across studies could be attributed to different initial plant densities since growth rates and biomass are dependent on plant density. In nutrient removal studies, higher initial frond densities resulted in decreased growth rates in *L. minor* [33,34] and decreased biomass and frond area in *Spirodela polyrrhiza* [35]. In contrast, Iqbal et al. [36] reported lower *Lemna minor* growth rates at low (25% cover) and high (100% cover) compared to moderate frond densities (50% cover), which they attributed to inverse density dependence and competition. High frond density was also associated with greater toxicity in plants. Dry weight decreased with increasing plant density in controls, and more so when plants were exposed to 20 mg/L Ni [37]. Similarly, increased initial frond density of *L. minor* and *S. polyrrhiza* resulted in decreased growth when exposed to the pesticide dimethomorph [38]. Färber and Kandeler [39,40] indicated the mechanism for decreased duckweed growth at higher frond densities is increased production of the growth inhibitor ethylene. When duckweeds become crowded and pushed together, this stimulates the production of ethylene which inhibits plant growth [39,40]. Therefore, initial frond density is important in growth and toxicological responses of duckweeds and should be considered in toxicity and phytoremediation studies.

We investigated how initial frond densities influence toxicity of AgNPs and AgNO_3_ of *L. minor* frond number, frond area, and dry weight. Most other works only evaluated frond number and/or dry weight. Total frond area is a good assessment of growth and biomass production and can be measured without biomass loss or injury to the plants. We assessed the effects on *Lemna minor* growth at three frond densities (20, 40, and 80 fronds per 28.5 cm^2^). Most studies only assessed toxicity on 21 or fewer fronds, and none have investigated the influence of frond density. We used AgNO_3_ to compare effects of AgNPs to ionic silver. AgNO_3_ readily provides dissolved silver ions [41] and is used to compare with AgNPs [25,26,27]. In most cases, AgNO_3_ had greater toxicity on duckweeds than AgNPs [25,27]. We hypothesized that AgNPs and AgNO_3_ would have decreased growth at higher initial frond densities, and AgNO_3_ would have greater inhibition overall. Frond density is not static, and laboratory experiments do not accurately reflect densities observed in the environment, so our goal was to assess the influence of frond density on silver toxicity.

## 2. Results

### 2.1. Experiment 1: Optimal Nutrient Concentration

Dilution of the Hoagland solution decreased growth. Plants in 2.5% Hoagland solution produced 1.5 times fewer fronds compared to 25% Hoagland solution, while plants in more diluted Hoagland solutions (0.25–1%) produced 2.4–4 times fewer fronds. There was no difference in dry weight and frond area between plants in 2.5% Hoagland solution and 25% Hoagland solution (Table 3). The 2.5% solution was similar in frond area and dry weight to the 1% Hoagland, but had greater frond area and dry weight than the 0.25–0.5% Hoagland solutions. 

### 2.2. Experiment 2: Effects of Silver Nanoparticle Concentrations

Dilution of the Hoagland solution resulted in greater AgNP stability. Zeta potential values of 2.5% Hoagland solution indicated stability (average ± standard deviation +25 ± 2 mV), while 25% Hoagland solution was unstable (+12 ± 1 mV). Plants exposed to 500 µg/L AgNPs had greater dry weight than plants exposed to 1000 µg/L, and had lower dry weight and frond area than plants exposed to no AgNPs (Table 4). The highest AgNP concentration (1000 µg/L) resulted in 38% inhibition of frond number and 75% inhibition of frond area. The next highest AgNP concentration (500 µg/L) resulted in 12% inhibition of frond number and 30% inhibition of frond area.

### 2.3. Experiment 3: Plant Growth Response of Lemna minor to Different Forms of Silver

**Figure 1:**
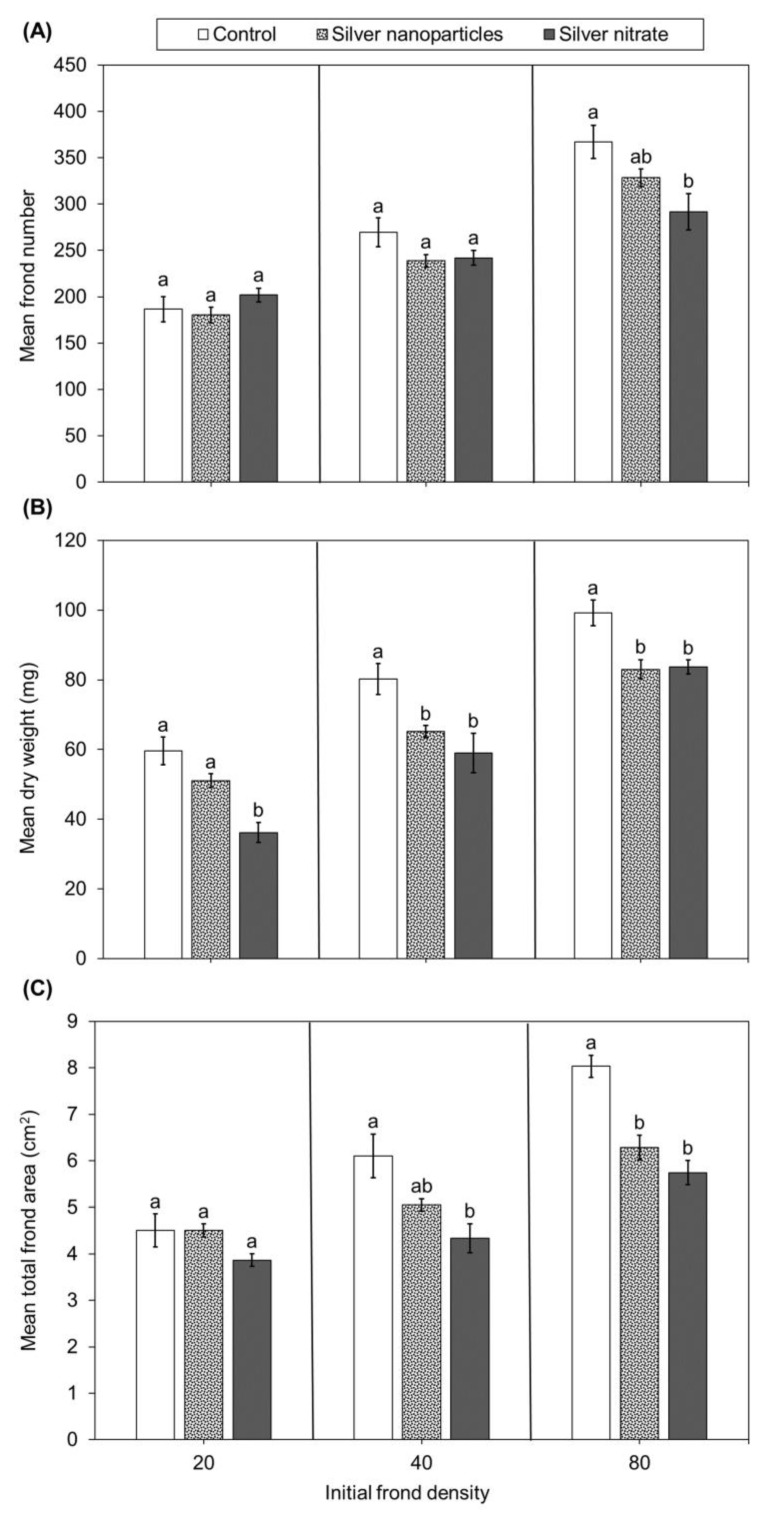
Mean ± standard error frond number (**A**), dry weight (**B**), and frond area (**C**) of *Lemna minor* after 14 days in control, 500 µg/L AgNPs, or 500 µg/L AgNO_3_ (*n* = 5) at different frond densities (20, 40, and 80 fronds per 28.5 cm^2^). Different letters indicate differences among treatments within initial frond density groups (one−way ANOVA, Tukey multiple comparisons tests, *p* < 0.05).

**Figure 2:**
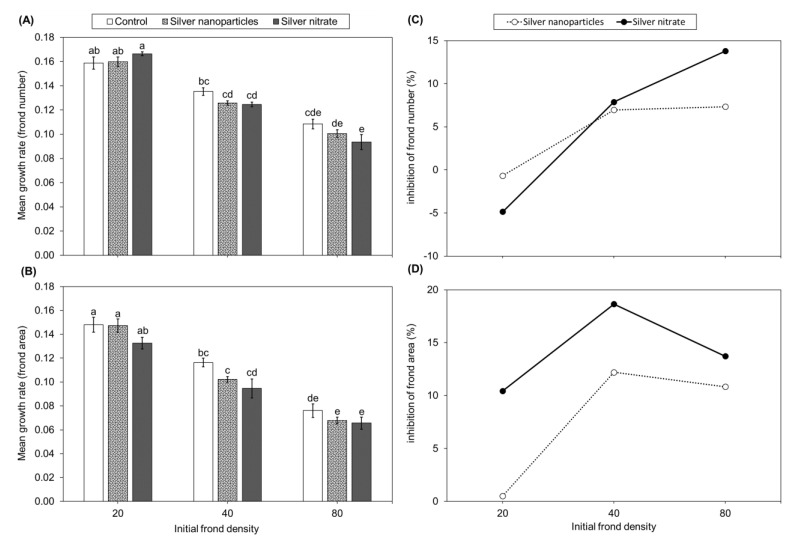
Mean ± standard error growth rate of frond number (**A**) and frond area (**B**) of *Lemna minor* after 14 days in control, 500 µg/L AgNPs, or 500 µg/L AgNO_3_ (*n* = 5) and percent inhibition of *Lemna minor* frond number (**C**) and frond area (**D**) at different frond densities (20, 40, and 80 fronds per 28.5 cm^2^). Different letters indicate differences among treatments and initial frond density groups (two-way ANOVA, Tukey multiple comparisons tests, *p* < 0.05).

Plants in the 80–AgNO_3_ produced fewer fronds than the 80–control, while plants in the 80–AgNP produced similar frond numbers as both the 80–control and 80–AgNO_3_ (Figure 1A). Plants in the 20 and 40 initial frond density groups (AgNP, AgNO_3_, and control) produced similar frond numbers among silver treatments and the control. Growth rates according to frond number decreased with increasing frond density regardless of silver treatment (Figure 2A). Percent inhibition of frond number increased with increased initial frond density (Figure 2C). Plants in the 40–AgNP, 40–AgNO_3_, 80–AgNP, and 80–AgNO_3_ experienced greater toxicity with 7–22% inhibition of frond numbers. Plants in the 20–AgNP experienced no inhibition of frond number and even stimulated plant growth in the 20–AgNO_3_.

Plants in the 20– and 40–AgNO_3_ had lower dry weights than the 20– and 40–control (Figure 1B). The 20–AgNO_3_ group also had lower dry weights compared to the 20–AgNP. The 80–AgNP and 80–AgNO_3_ had lower dry weights than the 80–control. The 40– and 80–AgNP had similar dry weights to the 40– and 80–AgNO_3_.

Plants in 80–AgNP and 80–AgNO_3_ groups had lower frond area than the 80–control (Figure 1C). Plants in 40–AgNO_3_ had lower frond area than the 40–control. Frond area growth rates decreased with increasing initial frond density (Figure 2B). Plants in 20– and 40–AgNP had higher growth rates than 80–AgNP. Plants in 40– and 80–AgNO_3_ had lower growth rates than 20–AgNO_3_. Plants in the 40 and 80 initial frond density groups of AgNP and AgNO_3_ experienced 11–19% inhibition of frond area (Figure 2D). Plants in 20–AgNO_3_ experienced 10% inhibition, while 20–AgNP experienced 1% inhibition.

**Figure 3:**
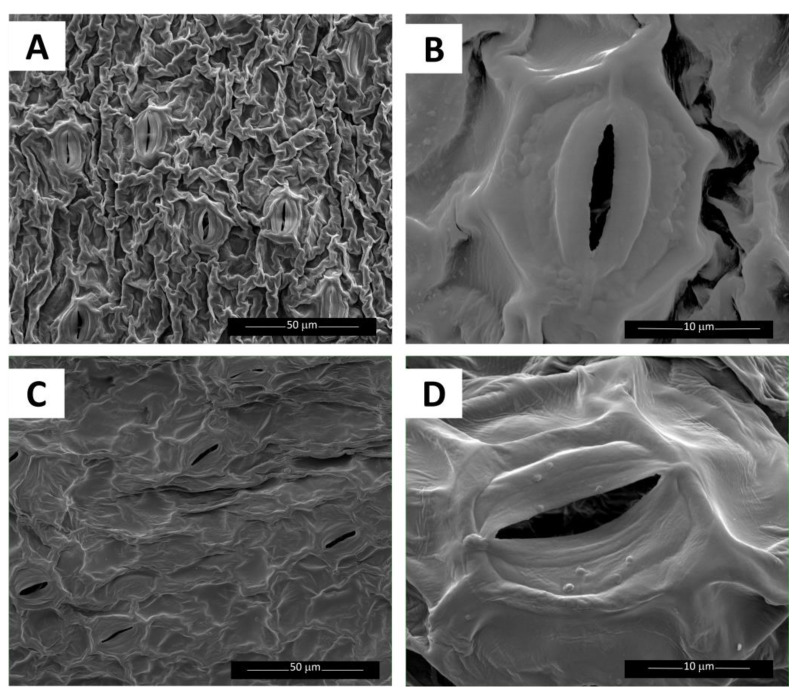
Scanning electron microscopy (SEM) images of *Lemna minor* fronds; upper leaf surface (**A**) and stomata (**B**) of 80–control; upper leaf surface (**C**) and stomata (**D**) of 80–AgNP.

SEM images showed that plants in 80–AgNP had different frond upper surface and stomata structure compared to 80–control (Figure 3). The frond surfaces were smoother, and the stomata and guard cells appeared flattened in 80–AgNP (Figure 3C,D).

## 3. Discussion

Previous work showed that dilution of standard growth media is required to maintain AgNP stability and prevent agglomeration in toxicity studies [12,24]. Gubbins et al. [24] used a 100× dilution growth media with a 2.6% reduction in growth rate to prevent agglomeration of AgNPs. Our study used a 10× dilution growth media with a 2.2% reduction in growth rate. Our dilution had no effect on dry weight and frond area while maintaining AgNP stability. Toxicity in *L. minor* varied with AgNP concentrations (5–10,000 μg/L) in other studies (Table 1). Our study used 500 μg/L AgNPs because it had sublethal effects on *L. minor* frond number and area, while smaller concentrations had similar effects as the controls or did not inhibit growth.

Overall, our study showed that *L. minor* biomass (dry weight) and growth rates are dependent on initial frond density regardless of silver exposure. At higher frond densities duckweeds became crowded in our study. Crowding results in plants being pushed together, leading to an increase in ethylene production, which in turn inhibits growth [39,40]. *Lemna minor* also experiences frond detachment and decreased growth rates in response to crowding [42]. Crowding can also result in decreased growth due to competition for limited nutrients, space, and light [36]. This means that plants at 20 and 80 initial frond densities do not have access to the same concentrations of nutrients and amounts of light and space. At high densities, more plants are competing for the same limited resources, which in turn will inhibit growth [35,36]. Growth inhibition is probably due to a combination of these effects of crowding in the controls and silver treatments in our study.

Plants exposed to silver at higher initial frond densities had lower dry weight and total frond area than the controls in the current study. Higher initial frond densities also resulted in increased inhibition of growth in the presence of silver. These findings were consistent with another study that reported increased inhibition due to Ni exposure with increasing duckweed density [37]. The inhibition of growth at higher densities could be due to the inhibitory effects of crowding [33,39,40] and competition for limited nutrients and space [35], while also experiencing the effects of silver toxicity. 

In the current study, growth rates (based on frond number and area) showed that silver had no effect when compared to the controls at the same initial frond densities. This was not consistent with findings of another study that reported increased initial *L. minor* and *S. polyrrhiza* densities resulted in lower growth rates in pesticide treatments than in controls [38]. Our growth rate findings indicated that the effects of crowding, and not the presence of silver, played a major role in inhibiting *L. minor* growth. Growth rates of our controls were consistent with other studies that reported decreased growth rates with increased initial duckweed density in the absence of a toxicant (i.e., controls) [33,34,35]. These effects on controls are probably due to the inhibitory effects of crowding, as mentioned previously [33,38].

AgNPs had no effect on frond number in our study, which coincided with Pereira et al. [25], who reported no effects on frond numbers per colony at similar AgNP concentrations to our study. In contrast, *L. minor* experienced reduced frond numbers after 7–14 days of exposure to AgNPs of similar size as in our study, but at 25–100 times lower concentrations (5–20 μg/L) than the AgNP concentrations we tested [22,23,24]. The differences in toxicity could be due to differences in initial frond density (5–21 fronds), growth media, nanoparticle synthesis, and AgNP stability.

AgNO_3_ decreased frond number in the highest initial frond densities while AgNPs had no effect. These findings are consistent with other studies that reported greater toxicity of AgNO_3_ than AgNPs on duckweeds [25,26,27,43]. AgNO_3_ resulted in decreased frond number, but at concentrations 4–100 times lower (5–120 µg/L) than the concentration in our study [24,25]. Souza et al. [26] also reported significant decreases in live fronds over time when *L. minor* was exposed to 1000 µg/L AgNO_3_. Ag^+^ ions are more readily available for plant uptake in AgNO_3_ than in AgNP treatments, which likely explains the greater toxicity of AgNO_3_ [26,41].

Our findings on AgNP effects on dry weight did not correspond with other studies. Some studies reported decreased dry weights, but at concentrations 50 times lower (10 µg/L) [24] and 16 times lower (32 µg/L) [23] than AgNP concentrations used in our study. However, Jiang et al. [27] and Ding et al. [31] reported reduced dry weights in duckweeds after exposure to 10–200 times higher AgNP concentrations than what we used. Reduction in dry weight in the highest frond densities of our study might be due to the inhibitory effects of overcrowding and competition [36,39]. Variations in AgNP toxicity could be due to differences in growth media and nanoparticle synthesis methods, which contributed to differences in nanoparticle stability. 

Similar to other studies, our dry weight findings emphasized that AgNO_3_ had greater toxic effects on plants than AgNPs. Jiang et al. [27] reported lower dry weight of *Spirodela polyrrhiza* when exposed to AgNO_3_ than those in the control and AgNP treatment. The sensitivity to AgNO_3_ at low and high initial frond densities also highlighted the greater toxicity of AgNO_3_ than AgNPs. At low densities, the greater toxic effect could be due to inverse density dependence [36], while at high densities the greater toxic effect could be due to the inhibitory effects of crowding [39,40].

Only one other study assessed the effects of AgNPs on frond area in duckweeds. Lalau et al. [20] reported no effects on *L. punctata* frond area at similar AgNP concentrations (1.3 times higher) to our study. They reported that frond area decreased in *L. punctata* at AgNP concentrations 21 times higher than in our study. This could be due to *L. punctata* having greater AgNP tolerance than *L. minor*. We observed reduced frond area, but not frond number for 80–AgNP plants, indicating that the toxic effect was due to decreased frond size. Again, these observations indicate that the inhibitory effects of overcrowding and competition possibly exacerbated AgNP toxicity at high initial frond densities. To further support our reasoning of inhibition due to overcrowding, higher densities (40 and 80) had decreased frond area when exposed to AgNO_3_.

Both forms of silver had no effect on growth rates based on frond number and area. Growth rates were only dependent on initial frond density, which coincides with other studies that reported that growth rates depended on initial plant density [33,34,35]. Final biomass (dry weight) was negatively affected in the silver treatments at higher initial plant densities (40 and 80). This indicates that silver probably affected frond thickness and root length, which were accounted for in dry weight (biomass) but were not accounted for in the growth rate measurements. Siddiqi and Husen [44] reported that AgNP interactions with proteins and carbohydrates influence total biomass and root and shoot growth. Inhibition of frond number and frond area at higher frond densities (40 and 80) further emphasized that frond density influenced the effect of silver toxicity. Lack of inhibition at low frond densities (20) could be due to the lack of crowding and pushing together of fronds, and the lack of competition for space, light, and nutrients, all of which could inhibit growth. Additionally, 20–AgNO_3_ plants produced more fronds than the 20–control plants, possibly because of the additional supply of nitrates from AgNO_3_.

SEM images provided some insight into potential mechanisms of inhibition at high frond densities. SEM images of the upper surface of the sample fronds from the 80–AgNP contrasted with findings reported by Lalau et al. [20]. Their control fronds had light undulations and a smoother appearance, while our control fronds had distinct undulations and a rough appearance. Lalau et al. [20] reported reduced stomatal openings in their AgNP samples. In our study, most stomata were open in both the AgNP and control samples, but the guard cells seemed less turgid, giving the structure a flatter appearance in AgNP samples than in the control. Opening and closing of stomata depend on the turgidity of guard cells. The stomata open when guard cells fill with water and become turgid. Reduced openings of the stomata can adversely affect photosynthesis, as the stomata allow for the movement of gases and water vapor [45]. Adverse effects on photosynthesis could help explain the inhibition observed at high frond densities.

Similar to our findings, several studies reported AgNPs reduced duckweed frond numbers, dry weight (biomass), and frond area [12,20,23,24]. However, most of these studies assessed the effects of AgNPs on small initial plant densities or biomass, that is, a small number of initial plants (10) or fronds (5–21), and none assessed the influence of initial frond density on AgNP toxicity. These studies also reported effects at low AgNP concentrations (<100 μg/L). However, the observed effects could be different with higher initial plant biomass exposed to these same AgNP concentrations because of crowding and possible dilution effects. Assessing the effects of nanoparticles on a small number of initial fronds, or on plants at a single low density, might limit the overall picture of the toxic effects of these chemicals on duckweeds. Additionally, observed effects could be different at higher initial plant biomass exposed to the same concentrations of nutrients and toxicants. Investigating the influence of different plant densities is important and applicable to what occurs in the environment, since duckweeds tend to form dense mats of mixed genera and species under suitable conditions [46]. The findings of the current study demonstrated that *Lemna* initial frond densities influence growth and their responses to silver, therefore initial and varying frond densities must be factored into toxicity tests and phytoremediation studies.

## 4. Materials and Methods

We collected *Lemna minor* plants from among *Wolffia brasiliensis* and *Spirodela polyrrhiza* growing in Valley Water Mill Pond, a spring impoundment in Springfield, Missouri. We identified *L. minor* using identification keys of Yatskievych [47] and Landolt [48]. Some defining characteristics that we used for identification included frond length (1–8 mm), frond description (flat, 3 veins, 1–2 times as long as wide), root length (greater than 3 cm), root description (one root per frond with rounded tip), color (fronds pale green on lower surface not reddish), and papillae on upper surface (not always visible) [47,48]. Voucher specimens were deposited at the Ozarks Regional Herbarium (SMS). Plants were triple rinsed with deionized water and placed in plastic totes containing three liters of 25% Hoagland solution to establish a culture. The 25% Hoagland solution contained: in m*M,* 0.5 KH_2_PO_4_, 1.5 KNO_3_, 0.5 MgSO_4_•7H_2_O, 0.25 NaCl, and in µ*M*, 11.5 H_3_BO_3_, 2.3 MnCl_2_, 0.19 ZnSO_4_, 0.08 CuS_4_, 0.026 H_2_MoO_4_, and 22.4 Fe-Na EDTA (as ferric-sodium EDTA complex) [49]. After adjusting the solution pH to 5.5–6, we added 1.25 m*M* Ca(NO_3_)_2_ and 2 m*M* HEPES. We used plants triple-rinsed with deionized water from this culture to conduct three experiments in unsterile conditions. In experiment 1, we tested effects of diluting Hoagland solution on growth. In experiment 2, we tested effects of different AgNP concentrations on growth. In experiment 3, we measured effects of AgNPs and AgNO_3_ on *L. minor* at different initial frond densities. All experiments were carried out in 170 mL acid-washed plastic cups on shelves with LED lighting in our laboratory (average temperature 23 °C, 14 h light: 10 h dark photoperiod, average light intensity 112 µmol m^−2^ s^−1^).

### 4.1. Experiment 1: Optimal Nutrient Concentration

**Figure 4:**
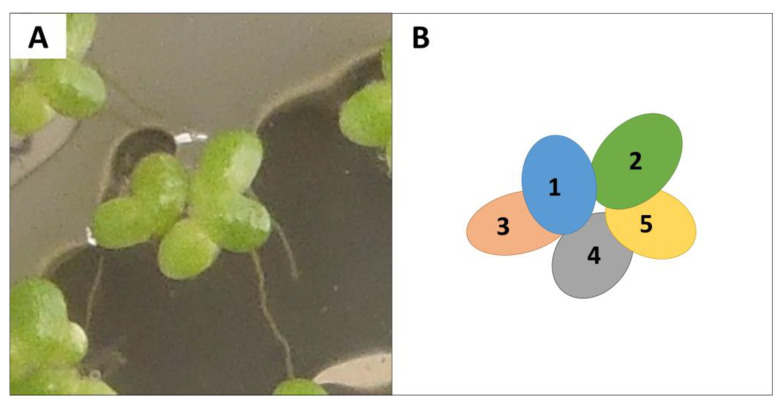
Image of a live *Lemna minor* colony (**A**) and diagram depicting how we differentiated and counted fronds (**B**).

Previous research recommended diluting nutrient solutions at pH 5.5 since AgNPs aggregate in standard nutrient solutions [12,24]. Dilution allows plants to be exposed to stable AgNP concentrations [24]. To determine the highest dilution that did not cause a relevant growth reduction, we conducted an experiment with *L. minor* in 170 mL plastic cups (40 fronds each) of 120 mL of 25%, 2.5%, 1%, 0.5%, 0.3%, and 0.25% Hoagland solution (5 replicates per dilution group). Figure 4 shows how fronds were identified and counted. Mean pH of Hoagland solutions remained relatively constant from beginning (5.58 ± 0.07) to end (5.50 ± 0.09). We took photos with a digital camera of each cup and a scale at the beginning, midpoint, and end of the 14-day experiment. These photos were used to determine frond number and area using Image J [50]. We counted and calculated area of live fronds, ignoring fronds that were white or more than 50% yellow [30] (Figure 4). Plants were collected, placed in glycine envelopes, and dried at 105 °C until constant weight. Based on this experiment, we used 2.5% Hoagland solution for subsequent experiments.

### 4.2. Experiment 2: Effects of Silver Nanoparticle Concentrations

To determine the lowest AgNP concentration that caused sub-lethal effects on *L. minor*, we conducted an AgNP range-finding experiment (PELCO^®^ 20 nm, silver nanospheres, citrate, Nanoexact^TM^ provided and evaluated by nanoComposix, Inc., San Diego, CA, USA). To assess nanoparticle stability, we measured the Zeta potential of 2.5% and 25% Hoagland solutions with 20 mg/L AgNPs using the Smoluchoski method on a NanoBrook Omini by Brookhaven Instruments Corporation, Holtsville, NY, USA. We transferred *L. minor* (40 fronds each) from our culture to 30 plastic cups containing 120 mL 2.5% Hoagland solution. Plants acclimatized to the solution for four days before addition of AgNPs. Each cup received AgNPs at concentrations of 0, 60, 125, 250, 500, or 1000 µg/L (five replicates per treatment group). After 14 days, we determined frond number, frond area, and dry weight as described above. Based on this experiment, we used 500 µg/L AgNPs or AgNO_3_ for experiment 3 because, at this concentration, AgNPs had moderate inhibition and sublethal effects on growth. Lower AgNP concentrations were either similar to controls or had little to no inhibition.

### 4.3. Experiment 3: Plant Growth Response of Lemna minor to Different Forms of Silver

Frond density refers to number of fronds per plastic cup (water surface area = 28.5 cm^2^) of 120 mL nutrient solution. This experiment used three initial frond densities (20, 40, and 80 fronds). *Lemna minor* were placed in plastic cups (15 cups per initial frond density group), each containing 120 mL 2.5% Hoagland solution. After four days of acclimatization, 500 µg/L AgNPs was added to five cups of each frond density group (20–AgNP, 40–AgNP, 80–AgNP) and 500 µg/L of AgNO_3_ to another five cups of each group (20–AgNO_3_, 40–AgNO_3_, 80–AgNO_3_). The remaining five cups from each frond density group received no silver and served as the controls (20–control, 40–control, 80–control). Plants grew for two weeks, and small volumes (<25 mL) of 2.5% Hoagland solution were added to cups twice a week to account for evaporation. After 14 days, we determined frond number, frond area, and dry weight as described above.

Frond surface and stomata aperture can be affected by exposure to AgNPs [20]. To evaluate the effects of AgNPs on frond morphology, at the time of harvest, we collected 3–4 colonies (2–4 fronds per colony) with roots from the 80–AgNP treatment and 80–control by first stirring and pulling out colonies for observation via a scanning electron microscope (SEM). The plants were carefully patted dry with paper towels, mounted, and coated using gold sputter with a coating thickness typically up to 20 nm, prior to visualization under FEI Quanta 200, a field emission gun (FEG) SEM using Secondary Electron (SE) and Back-scattered (BSE) imaging. The SEM was operated under an accelerating voltage of 20 kV and a high vacuum mode with a chamber pressure below 1 × 10^−5^ Torr.

### 4.4. Calculations and Statistical Analysis

We calculated means and standard error for frond number, dry weight, and frond area for each experiment. We calculated specific growth rates for frond number and area for each replicate of the control and treatments using Equation (1) [30].

(1) $$\mu_{i-j}=\frac{\ln\left(N_{j}\right)-\ln\left(N_{i}\right)}{t}$$

where:

*μ_i–j_* = average specific growth rate from time *i* to *j*
*Ni* = measurement in treatment or control at time *i*
*Nj* = measurement in treatment or control at time *j*
*t* = time from *i* to *j*

We calculated percent inhibition of growth rate (*I_r_*) for each treatment group using Equation (2) [30].

(2) $$\%I_{r}=\frac{\left(\mu C-\;\mu T\right)}{\mu C}\times 100$$

where:

μ*C* = mean growth rate of control
μ*T* = mean growth rate of treatment

Significance of differences was determined by One–Way or Two–Way ANOVA (α = 0.05) followed by Tukey multiple comparisons tests (α = 0.05) in Minitab^®^ 19. For experiments 1 and 2, we analyzed for differences in frond number, frond area, and dry weight between Hoagland solutions or AgNPs. We analyzed for differences in frond number, frond area, dry weight, and growth rate among AgNPs and AgNO_3_ treatments and the control for each initial frond density group. Prior to statistical analyses, we transformed data not meeting assumptions of ANOVA using Johnson transformation to achieve homogeneity of variance and normal distributions.

## 5. Conclusions

We evaluated the toxic effects of AgNPs and AgNO_3_ on *L. minor* growth at different initial plant densities (20, 40, 80 fronds per 28.5 cm^2^). Greater inhibition and decreased biomass (dry weight) at higher frond densities (40 and 80) in silver treatments than in controls indicated that increasing frond density exacerbated silver toxicity. Decreased growth rates with increasing initial frond density, regardless of the presence of silver, emphasized that the effects of crowding played a major role in growth inhibition. Future work should assess the influence of different plant densities on the toxicity of nanoparticles and other contaminants, since overcrowding could play a role in the toxicity effect. In addition, since both forms of silver affected frond number, dry weight, and frond area differently, it is important to assess the toxic effects of contaminants on multiple growth parameters in toxicity studies.

## Figures and Tables

**Table 1:** Toxicity effects of silver nanoparticles (AgNP) of varying size on floating species of the family Araceae (AgNP concentrations listed indicate the lowest concentrations with an observed effect).

Plant Species	AgNP (µg/L)	Size (nm)	Effect	References
*Landoltia punctata*	10	19–27	Decreased growth rate, frond area, and root size	[20]
	10	40–60	Increased reactive oxygen species (ROS)	[21]
*Lemna gibba*	10	50	Decreased frond number	[12]
	100		Decreased cell viability	
*Lemna minor*	5	10–80	Decreased frond number	[22]
	8	5–20	Decreased frond number and dry weight	[23]
	10	20	Decreased dry weight	[24]
	20		Decreased frond number	
	50	79	Decreased growth rate	[25]
	800		Decreased fronds per colony	
	2000		Chlorosis	
	10,000	30	Decreased growth rate	[26]
*Spirodela polyrrhiza*	500	6	Chlorosis	[27]
	1000		Root abscission	
	5000		Decreased dry weight	
	10,000	20	Increased ROS	[28]
*Wolffia globosa*	1000	10	Decreased photosynthetic activity	[29]
	10,000		Decreased photosynthetic pigments	

**Table 2:** Initial frond numbers and growth measurements used in studies involving silver nanoparticles and duckweed species (x indicates that the growth measurement was evaluated in the referenced study).

Plant Species	Initial Frond Number	Growth Measurements			References
		Frond Number	Dry Weight	Frond Area	
*Landoltia punctata*	not given	x		x	[20]
*Lemna gibba*	15	x			[12]
*Lemna minor*	10	x			[25]
	15–20	x	x		[24]
	21	x	x		[23]
	not given		x		[31]
	not given	x			[22]
*Lemna aequinoctialis (formerly Lemna paucicostata)*	5	x			[32]
*Spirodela polyrrhiza*	not given	x	x		[27]

**Table 3:** Mean ± standard error for Experiment 1 dry weight, frond number, and frond area of *Lemna minor* after 14 days in different Hoagland solutions.

Hoagland Nutrient Solution	Frond Number		Dry Weight (mg)		Frond Area (cm^2^)	
25%	471 ± 12	a	121 ± 3	a	27 ± 2	a
2.5%	307 ± 11	b	83 ± 3	ab	16 ± 0.7	ab
1%	193 ± 2	c	55 ± 5	bc	8 ± 0.3	b
0.5%	148 ± 2	d	43 ± 3	cd	6 ± 0.3	c
0.3%	132 ± 4	d	37 ± 2	cd	5 ± 0.2	c
0.25%	115 ± 2	e	32 ± 4	d	5 ± 0.4	c

Different letters indicate differences between Hoagland solutions for each growth variable (One–way ANOVA, followed by Tukey Multiple Comparison tests, *p* < 0.05, *n* = 5).

**Table 4:** Mean ± standard error for Experiment 2 dry weight, frond number, frond area, and percent inhibition (frond number and area) of *Lemna minor* after 14 days in different silver nanoparticle (AgNP) concentrations.

AgNP (μg/L)	Dry Weight (mg)		Frond Number		Frond Area (cm^2^)		% Inhibition of Frond Number	% Inhibition of Frond Area
0	78 ± 2	a	208 ± 13	ab	7 ± 0.4	a	0	0
60	72 ± 0.6	b	220 ± 10	ab	6 ± 0.2	ab	−4	6
125	69 ± 2	b	236 ± 14	a	6 ± 0.3	a	−8	9
250	67 ± 0.2	bc	200 ± 7	ab	6 ± 0.4	ab	2	12
500	61 ± 1	c	169 ± 9	bc	4 ± 0.4	b	12	30
1000	53 ± 1	d	111 ± 10	c	2 ± 0.3	c	38	75

Different letters indicate differences between AgNP concentrations for each growth variable (One–way ANOVA, followed by Tukey Multiple Comparison tests, *p* < 0.05, *n* = 5).

## Data Availability

Data, metadata, and calculation tools are available in the manuscript or upon request from the corresponding author (lkissoon@missouristate.edu).

## References

[1] Jain D., Daima H.K., Kachhwaha S., Kothari S.L. (2009). Synthesis of plant-mediated silver nanoparticles using papaya fruit extract and evaluation of their anti microbial activities. Dig. J. Nanomater. Biostruct..

[2] Zhang X.F., Liu Z.G., Shen W., Gurunathan S. (2016). Silver nanoparticles: Synthesis, characterization, properties, applications, and therapeutic approaches. Int. J. Mol. Sci..

[3] Choi O., Deng K.K., Kim N.-J., Ross L., Surampalli R.Y., Hu Z. (2008). The inhibitory effects of silver nanoparticles, silver ions, and silver chloride colloids on microbial growth. Water Res..

[4] Choi O.K., Hu Z.Q. (2009). Nitrification inhibition by silver nanoparticles. Water Sci. Technol..

[5] Benn T.M., Westerhoff P. (2008). Nanoparticle silver released into water from commercially available sock fabrics. Environ. Sci. Technol..

[6] Geranio L., Heuberger M., Nowack B. (2009). The behavior of silver nanotextiles during washing. Environ. Sci. Technol..

[7] Mueller N.C., Nowack B. (2008). Exposure modeling of engineered nanoparticles in the environment. Environ. Sci. Technol..

[8] Blaser S.A., Scheringer M., MacLeod M., Hungerbühler K. (2008). Estimation of cumulative aquatic exposure and risk due to silver: Contribution of nano-functionalized plastics and textiles. Sci. Total Environ..

[9] Kaegi R., Sinnet B., Zuleeg S., Hagendorfer H., Mueller E., Vonbank R., Boller M., Burkhardt M. (2010). Release of silver nanoparticles from outdoor facades. Environ. Pollut..

[10] Kaegi R., Voegelin A., Ort C., Sinnet B., Thalmann B., Krismer J., Hagendorfer H., Elumelu M., Mueller E. (2013). Fate and transformation of silver nanoparticles in urban wastewater systems. Water Res..

[11] Lee W.-M., Kwak J.I., An Y.-J. (2012). Effect of silver nanoparticles in crop plants *Phaseolus radiatus* and *Sorghum bicolor*: Media effect on phytotoxicity. Chemosphere.

[12] Oukarroum A., Barhoumi L., Pirastru L., Dewez D. (2013). Silver nanoparticle toxicity effect on growth and cellular viability of the aquatic plant *Lemna gibba*. Environ. Toxicol. Chem..

[13] Thwala M., Klaine S., Musee N. (2021). Exposure Media and Nanoparticle Size Influence on the Fate, Bioaccumulation, and Toxicity of Silver Nanoparticles to Higher Plant *Salvinia minima*. Molecules.

[14] Yin L., Colman B.P., McGill B.M., Wright J.P., Bernhardt E.S. (2012). Effects of silver nanoparticle exposure on germination and early growth of eleven wetland plants. PLoS ONE.

[15] Naumann B., Eberius M., Appenroth K.-J. (2007). Growth rate based dose–response relationships and EC-values of ten heavy metals using the duckweed growth inhibition test (ISO 20079) with *Lemna minor* L. clone St. J. Plant Physiol..

[16] Daud M.K., Shafaqaat A., Abbas Z., Zaheer I.E., Riaz M.A., Malik A., Hussain A., Rizwan M., Zia-ur-Rehman M., Zhu S.J. (2018). Potential of Duckweed (*Lemna minor*) for the Phytoremediation of Landfill Leachate. J. Chem..

[17] Ekperusi A.O., Sikoki F.D., Nwachukwu E.O. (2019). Application of common duckweed (*Lemna minor*) in phytoremediation of chemicals in the environment: State and future perspective. Chemosphere.

[18] Chaudhary E., Sharma P. (2019). Chromium and cadmium removal from wastewater using duckweed—*Lemna gibba* L. and ultrastructural deformation due to metal toxicity. Int. J. Phytoremediat..

[19] Landesman L., Fedler C., Duan R., Ansart A.A., Gill S.S., Lanza G.R., Rast W. (2010). Plant Nutrient Phytoremediation Using Duckweed. Eutrophication: Causes, Consequences and Control.

[20] Lalau C.M., Simioni C., Vicentini D.S., Ouriques L.C., Mohedano R.A., Puerari R.C., Matias W.G. (2020). Toxicological effects of AgNPs on duckweed (*Landoltia punctata*). Sci. Total Environ..

[21] Thwala M., Musee N., Sikhwivhilu L., Wepener V. (2013). The oxidative toxicity of Ag and ZnO nanoparticles towards the aquatic plant *Spirodela punctata* and the role of testing media parameters. Environ. Sci. Process Impacts.

[22] Minogiannis P., Valenti M., Kati V., Kalantzi O.-I., Biskos G. (2019). Toxicity of pure silver nanoparticles produced by spark ablation on the aquatic plant *Lemna minor*. J. Aerosol. Sci..

[23] Üçüncü E., Özkan A.D., Kurşungöz C., Ülger Z.E., Ölmez T.T., Tekinay T., Ortaç B., Tunca E. (2014). Effects of laser ablated silver nanoparticles on *Lemna minor*. Chemosphere.

[24] Gubbins E.J., Batty L.C., Lead J.R. (2011). Phytotoxicity of silver nanoparticles to *Lemna minor* L. Environ. Pollut..

[25] Pereira S.P.P., Jesus F., Aguiar S., de Oliveira R., Fernandes M., Ranville J., Nogueira A.J.A. (2018). Phytotoxicity of silver nanoparticles to *Lemna minor*: Surface coating and exposure period-related effects. Sci. Total Environ..

[26] Souza L.R., Corrêa T.Z., Bruni A.T., da Veiga M.A. (2021). The effects of solubility of silver nanoparticles, accumulation, and toxicity to the aquatic plant *Lemna minor*. Environ. Sci. Pollut. Res..

[27] Jiang H.-S., Li M., Chang F.-Y., Li W., Yin L.-Y. (2012). Physiological analysis of silver nanoparticles and AgNO_3_ toxicity to *Spirodela polyrhiza*. Environ. Toxicol. Chem..

[28] Jiang H.-S., Qiu X.-N., Li G.-B., Li W., Yin L.-Y. (2014). Silver nanoparticles induced accumulation of reactive oxygen species and alteration of antioxidant systems in the aquatic plant *Spirodela polyrhiza*. Environ. Toxicol. Chem..

[29] Zou X., Li P., Huang Q., Zhang H. (2016). The different response mechanisms of *Wolffia globosa*: Light-induced silver nanoparticle toxicity. Aquat. Toxicol..

[30] Organisation for Economic Co-operation and Development (2006). OECD Guidelines for the Testing of Chemicals, Section 2. Effects on Biotic Systems, Test No. 221: Lemna sp. Growth Inhibition Test.

[31] Ding Y., Bai X., Ye Z., Gong D., Cao J., Hua Z. (2019). Humic acid regulation of the environmental behavior and phytotoxicity of silver nanoparticles to *Lemna minor*. Environ. Sci. Nano.

[32] Kim E., Kim S.-H., Kim H.-C., Lee S.G., Lee S.J., Jeong S.W. (2011). Growth inhibition of aquatic plant caused by silver and titanium oxide nanoparticles. Toxicol. Environ Health Sci..

[33] Frédéric M., Samir L., Louise M., Abdelkrim A. (2006). Comprehensive modeling of mat density effect on duckweed (*Lemna minor*) growth under controlled eutrophication. Water Res..

[34] Walsh É., Coughlan N.E., O’Brien S., Jansen M.A.K., Kuehnhold H. (2021). Density Dependence Influences the Efficacy of Wastewater Remediation by *Lemna minor*. Plants.

[35] Zhang L.M., Jin Y., Yao S.M., Lei N.F., Chen J.S., Zhang Q., Yu F.H. (2020). Growth and morphological responses of duckweed to clonal fragmentation, nutrient availability, and population density. Front. Plant. Sci..

[36] Iqbal J., Javed A., Javed H. (2021). Effect of Initial Plant Density on Growth and Nutrients Removal Efficiency of Duckweed (*Lemna minor*) from Leachate. Asian J. Environ. Ecol..

[37] Demirezen D., Aksoy A., Uruç K. (2007). Effect of population density on growth, biomass and nickel accumulation capacity of *Lemna gibba* (Lemnaceae). Chemosphere.

[38] Dosnon-Olette R., Couderchet M., El Arfaoui A., Stéphanie S., Philippe E. (2010). Influence of initial pesticide concentrations and plant population density on dimethomorph toxicity and removal by two duckweed species. Sci. Total Environ..

[39] Färber E., Kandeler R. (1989). Significance of Calcium Ions in the Overcrowding Effect in Spirodela polyrrhiza P 143. J. Plant Physiol..

[40] Färber E., Kandeler R. (1990). Phytochrome effect on the ethylene production after overcrowding in *Spirodela* (Lemnaceae). Phyton.

[41] Stegemeier J.P., Colman B.P., Schwab F., Wiesner M.R., Lowry G.V. (2017). Uptake and distribution of silver in the aquatic plant *Landoltia punctata* (duckweed) exposed to silver and silver sulfide nanoparticles. Environ. Sci. Technol..

[42] Kufel L., Strzałek M., Przetakiewicz A. (2018). Plant response to overcrowding—*Lemna minor* example. Acta Oecologica.

[43] Iannelli M.A., Bellini A., Venditti I., Casentini B., Battocchio C., Scalici M., Ceschin S. (2022). Differential phytotoxic effect of silver nitrate (AgNO_3_) and bifunctionalized silver nanoparticles (AgNPs-Cit-L-Cys) on *Lemna* plants (duckweeds). Aquat. Toxicol..

[44] Siddiqi K.S., Husen A. (2022). Plant response to silver nanoparticles: A critical review. Crit. Rev. Biotechnol..

[45] Raven P.H., Evert R.F., Eichorn S.E. (1992). Biology of Plants.

[46] Reimer D.N. (1984). Introduction to Freshwater Vegetation.

[47] Yatskievych G. (1999). Steyermark’s Flora of Missouri, Revised ed..

[48] Landolt E., Flora of North America Editorial Committee (2000). Lemnaceae. Flora of North America.

[49] Zayed A., Gowthaman S., Terry N. (1998). Phytoaccumulation of trace elements by wetland plants: I. Duckweed J. Environ. Qual..

[50] Schneider C.A., Rasband W.S., Eliceiri K.W. (2012). NIH Image to ImageJ: 25 years of image analysis. Nat. Methods.

